# Antagonistic Control of Genetic Circuit Performance for Rapid Analysis of Targeted Enzyme Activity in Living Cells

**DOI:** 10.3389/fmolb.2020.599878

**Published:** 2021-01-12

**Authors:** Kil Koang Kwon, Haseong Kim, Soo-Jin Yeom, Eugene Rha, Jinju Lee, Hyewon Lee, Dae-Hee Lee, Seung-Goo Lee

**Affiliations:** ^1^Synthetic Biology and Bioengineering Research Center, Korea Research Institute of Bioscience and Biotechnology (KRIBB), Daejeon, South Korea; ^2^Department of Biosystems and Bioengineering, KRIBB School of Biotechnology, University of Science and Technology (UST), Daejeon, South Korea; ^3^School of Biological Sciences and Technology, Chonnam National University, Gwangju, South Korea

**Keywords:** inhibitor, antagonist, genetic circuit, phenolic compound, flow cytometry, resistor

## Abstract

Genetic circuits have been developed for quantitative measurement of enzyme activity, metabolic engineering of strain development, and dynamic regulation of microbial cells. A genetic circuit consists of several bio-elements, including enzymes and regulatory cassettes, that can generate the desired output signal, which is then used as a precise criterion for enzyme screening and engineering. Antagonists and inhibitors are small molecules with inhibitory effects on regulators and enzymes, respectively. In this study, an antagonist and an inhibitor were applied to a genetic circuit for a dynamic detection range. We developed a genetic circuit relying on regulators and enzymes, allowing for straightforward control of its output signal without additional genetic modification. We used *para*-nitrophenol and alanine as an antagonist of DmpR and inhibitor of tyrosine phenol-lyase, respectively. We show that the antagonist resets the detection range of the genetic circuit similarly to a resistor in an electrical logic circuit. These biological resistors in genetic circuits can be used as a rapid and precise controller of variable outputs with minimal circuit configuration.

## Introduction

Designing a genetic circuit is a crucial step for programming of living organisms, a long-term aim of synthetic biology (Brophy and Voigt, 2014; Nielsen et al., 2016). Transcriptional regulators in the circuits can control downstream gene expression by recognizing and transmitting specific input signals (Eggeling et al., 2015; Mahr and Frunzke, 2016). Applications include metabolic flux regulation in metabolic engineering (Rogers et al., 2015) and circuit-based high-throughput enzyme screening (Choi et al., 2014). Of these, genetic circuits for screening enzymes have greatly evolved over the last decade by employing various transcriptional regulators including amidase (BenR), alcohol dehydrogenase (SoxR), phosphatase (DmpR), and lactam synthase (NitR) (Uchiyama and Miyazaki, 2010; Choi et al., 2014; Siedler et al., 2014; Yeom et al., 2018).

Fine tuning of gene expression is also necessary to enable complex and precise control of biological reactions. This fine tuning can be achieved by adjusting the output signal range of the genetic elements (Brophy and Voigt, 2014; Smanski et al., 2014). Despite significant advances in the engineering of tools for genetics, the development of efficient genetic circuits remains a time-consuming and labor-intensive process involving repetitive trial and error. In addition, synthetic circuits offset their sensitivity to balance the expression of regulator and reporter genes, which can lead to the loss of flexibility under various environmental conditions (Hausser et al., 2019). A highly sensitive genetic circuit is advantageous for detecting minute clues regarding enzyme activity from a natural sample or metagenome (Ngara and Zhang, 2018; Markel et al., 2020). However, once a genetic circuit reaches its maximum dynamic range for detecting the enhanced activity of engineered enzymes, it is essential to use another module with an appropriate operational range to further engineer enzyme activity. Therefore, like variable resistors in electric circuits, genetic circuit resistors (GCRs) should be developed to control the dynamic range of genetic circuits.

Inhibitors in enzymatic reactions and antagonists in allosteric proteins, such as transcriptional regulators, are molecules that act as negative feedback elements for each activity (Dixon, 1953; Kenakin, 2007). These molecules can bind to the active site of proteins and decrease their activity by interfering with enzyme-substrate or regulator-ligand complex formation. This property can be used to control the sensitivity and dynamic range of a genetic circuit by simply adding small molecules to the reaction system (Xie et al., 2014). By adjusting the concentration of the small molecules, they can be used as variable resistors for tunable signal production. These biological resistors may be implemented for switchable and precise monitoring of enzyme activity, while the genetic circuit maintains hypersensitivity without additional genetic modification.

We previously reported a genetic enzyme screening system (GESS) consisting of DmpR as a phenol-dependent transcriptional regulator and green fluorescent protein (GFP) as a reporter protein (Choi et al., 2014; Kwon et al., 2020). The quantitative range of a genetic circuit relying on an enzyme and regulator could be adjusted by using an enzyme-inhibitor pair and regulator-antagonist pair. As a proof of concept, DmpR-GESS and tyrosine phenol-lyase (TPL), which produces phenol from a tyrosine substrate, were used to model a genetic circuit and enzyme. Alanine and *para*-nitrophenol (*p*NP) were used as the enzyme inhibitor and antagonist of DmpR, respectively. We controlled the output of the genetic circuit using elements related to the enzyme and regulator without genetic modification. In various applications, such as enzyme evolution, the circuit could be controlled using bio-parts and related inhibitors or antagonists as GCRs.

## Materials and Methods

### Materials

All chemicals were purchased from Sigma-Aldrich (St. Louis, MO, USA). DNA polymerase and Gibson assembly kits were purchased from New England Biolabs (Ipswich, MA, USA). All oligonucleotides were synthesized by Macrogen (Daejeon, Korea). Plasmid DNA isolation and DNA extraction from agarose gels were performed using Qiagen kits (Hilden, Germany). DNA preparation and related techniques were performed according to the manufacturer's protocols.

### Strains and Plasmids

*Escherichia coli* DH5α was used for cloning and genetic circuit experiments. Plasmids, pDmpR-GESS and pmDmpR-GESS were obtained from previous studies (Choi et al., 2014). The TPL gene from *Citrobacter freundii* and pAR plasmids (Kim et al., 2017) were amplified by PCR (TPL forward primer: 5′-TCA GCA GGA TCA CCA TAT GAA TTA TCC GGC AGA-3′, TPL reverse primer: 5′-TTG CGT TGC GCT TAG CTT TAG ATA TAG TCA AAG C-3′, pAR forward primer: 5′-GCT TTG ACT ATA TCT AAA GCT AAG CGC AAC GCA A-3′, pAR reverse primer: 5′-TCT GCC GGA TAA TTC ATA TGG TGA TCC TGC TGA A-3′). DNA fragments purified by agarose gel elution were ligated by Gibson assembly, and then transformed into DH5α cells to construct the pAR-TPL plasmid.

### Analysis of Regulator-Antagonist Output Signal

Cells harboring pDmpR-GESS or pmDmpR-GESS were cultivated in lysogeny broth (LB) medium (10 g tryptone, 5 g yeast extract, and 5 g NaCl per liter) and M9 minimal medium (12.8 g Na_2_HPO_4_·7H_2_O, 3 g KH_2_PO_4_, 0.5 g NaCl, 1 g NH_4_Cl, 2 mM MgSO_4_, 0.1 mM CaCl_2_, and 0.01% (w/v) thiamine per later) supplemented with 4 g/L acetate as a carbon source and 50 μg/mL ampicillin. For the two-step phenol reaction, the cells were grown in LB at 37°C until an OD_600_ of 2.0 was reached, and then the culture media was changed to fresh M9 with 1 mM aromatic compounds and various concentrations of phenol by mild centrifugation (1,000 × g, 5 min) (Kwon et al., 2020). After 15 h of incubation at 37°C, the fluorescence intensities of the cells were measured using a FACSAriaIII (BD Biosciences, Franklin Lakes, NJ, USA) with a blue laser source (488 nm) and an FL1 (530/30 nm) photomultiplier tube. Data were acquired using BD CellQuest Pro (version 4.0.2, BD Biosciences) and analyzed using Flowjo software (Flowjo, Ashland, OR, USA).

To examine the antagonistic effect of *p*NP, cells harboring pDmpR-GESS were cultured in a two-step reaction in the presence of various concentrations of phenol and *p*NP. Fluorescence intensity and the optical density at 600 nm (OD_600_) were analyzed with a multi-label reader (Victor V, PerkinElmer, Waltham, MA, USA).

### Analysis of Enzyme Inhibitor Output Signals

To detect enzymatic activity related to inhibition, cells harboring DmpR-GESS and the TPL gene were cultured in a two-step reaction. After replacing the M9 media with 1 mM tyrosine, 10 μM pyridoxal 5′-phosphate (PLP), and various concentrations of alanine, cell growth, and fluorescence intensities were measured with a FACSAriaIII and an Infinite 200 PRO microplate reader (Tecan, Mãnnedorf, Switzerland).

To detect enzymatic activity arising from the antagonistic effect of *p*NP, cells harboring pDmpR-GESS and the TPL gene were grown in LB with 20 μM L-rhamnose, 10 μM PLP, 50 μg/mL ampicillin, and 25 μg/mL chloramphenicol. After replacing the M9 media with 1 mM tyrosine, 10 μM PLP, and various concentrations of *p*NP, cell growth, and fluorescence intensity were measured with a microplate reader.

To detect the inhibitory effect of alanine in the solid phase, cells harboring TPL and the genetic circuit were cultured on LB agar plates with 1 mM tyrosine, 10 μM PLP, and 1 mM alanine at 37°C for 20 h. Fluorescence images were acquired using a fluorescence microscope (AZ100M, Nikon, Tokyo, Japan) with epifluorescence and diascopic DIC accessories. Images were acquired with a monochrome CCD camera (DS-Qi1Mc, Nikon) using a fluorescence filter set (GFP-HQ, Nikon) (Ex 455–485 nm, DM 495, BA 500–545). Images were processed and analyzed using Nikon's NIS-Elements AR 4.2 software.

## Results and Discussion

### Antagonistic Ligand-Dependent Sensitivity Control in a Genetic Circuit

**Figure 1:**
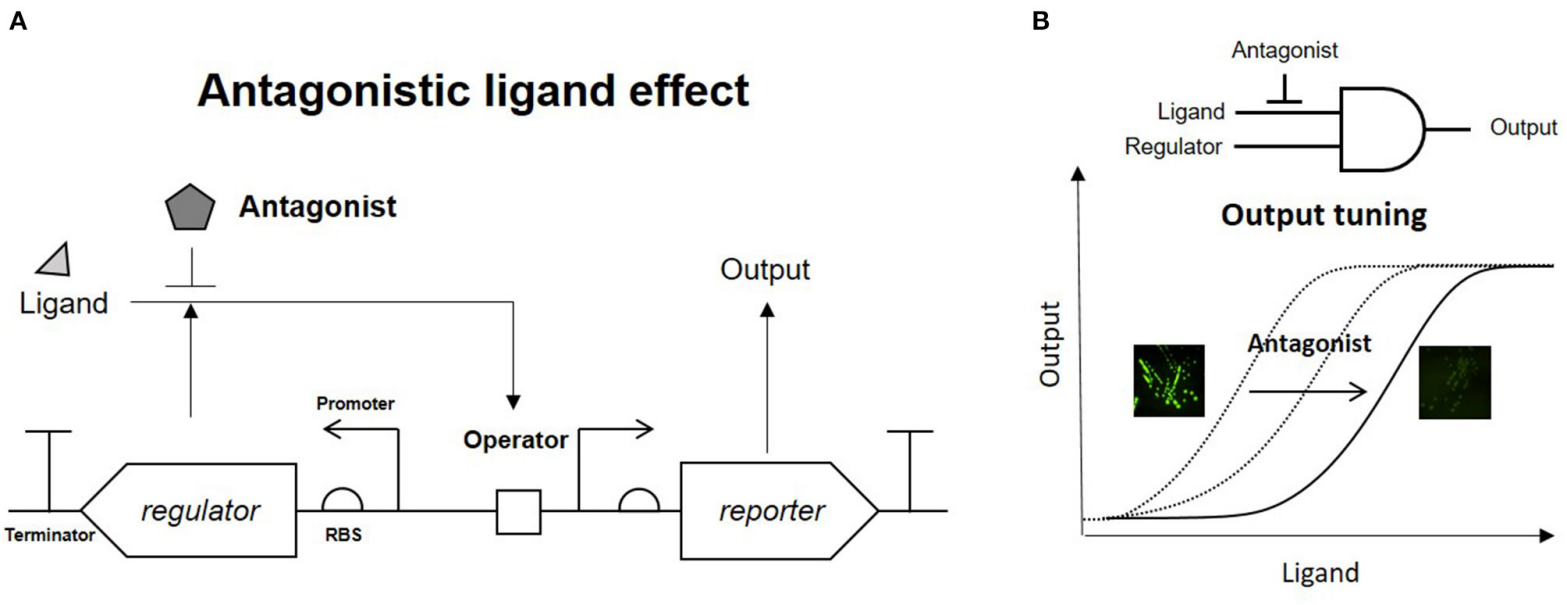
Strategy of antagonistic ligand-dependent sensitivity control in a genetic circuit. **(A)** Genetic circuit configuration using regulator and antagonist. **(B)** Logic gate and output control effect of antagonist.

A phenol-responsive genetic circuit used as a DmpR-GESS consists of a regulator and ligand that form an AND logic gate. An AND logic gate recognizes two inputs simultaneously (Tabor et al., 2009). Our circuit is composed of two AND logic gates: the first logic gate has two inputs, the enzyme and its substrate, and the subsequent logic gate uses the output from the first AND gate and regulator as its inputs. Addition of an enzyme inhibitor and a regulator antagonist can act as resistors for the two AND gates. Figure 1A shows the strategy of the association between the antagonist and regulator in the genetic circuit. The antagonistic ligand, which binds to DmpR and inhibits transcriptional initiation, can be used as a GCR to suppress the output signal. In addition, the GCR can modulate the dynamic detection range of the genetic circuit by simple addition without genetic modification (Figure 1B).

Wild-type (WT) DmpR responds to phenol, triggering downstream expression of reporter genes. However, *p*NP does not affect WT DmpR activity, but only induces transcriptional initiation when a single point mutation of E135K is inserted into the WT DmpR (Supplementary Figure 1; Choi et al., 2014). The crystal structure of DmpR was recently solved and was found to possess a binding pocket containing Glu135 at a distance from the phenol binding site (Supplementary Figure 2; Park et al., 2020). The E135K mutation of DmpR may affect the position of Arg36, thus inducing a conformational change in the sensory domain dimer and activating transcriptional initiation. As *p*NP can bind to WT DmpR naturally without inducing transcriptional initiation, it may act as an antagonist.

**Figure 2:**
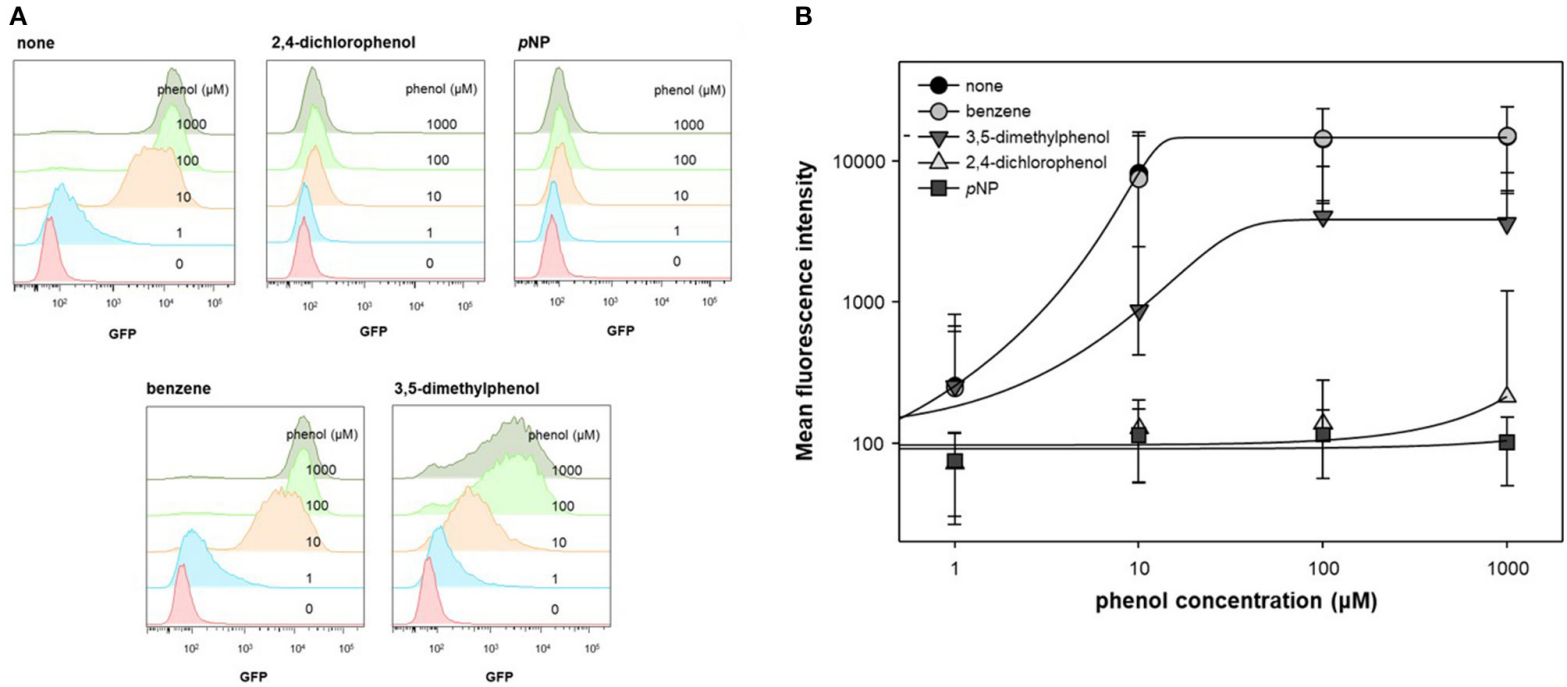
Cell population level analysis of antagonist ligand effect. **(A)** Flow cytometry fluorescence profile was analyzed using a DmpR-based genetic circuit with putative antagonists of DmpR and phenol. Aromatic compounds at 1 mM were added to the reaction. **(B)** Data recalculation from fluorescence profiles using Flowjo software. Values are presented as the median and robust standard deviation (rSD) of each histogram.

In previous studies, *ortho*- substituted phenolic compounds were shown to strongly induce DmpR-based transcriptional initiation (O'Neill et al., 1999; Choi et al., 2014). In contrast, some *meta*- or *para*- substituted phenolic compounds do not activate DmpR, even when inhibiting its ATPase activity. In this experiment, benzene, 3,5-dimethylphenol, 2,4-dichlorophenol, and *p*NP were administered along with phenol to test the inhibitory effect of DmpR. As DmpR is a σ54-dependent transcriptional regulator, it responds to metabolism of alternative carbon sources such as acetate (Kwon et al., 2020). To activate DmpR in the genetic circuit, cells harboring pDmpR-GESS were cultured in LB at and the culture media were changed to fresh M9 media containing 4 g/L acetate, 1 mM aromatic compounds, and various concentrations of phenol. Fluorescence intensity at a single-cell level was measured to evaluate the antagonist effect by flow cytometry (Figure 2A). Benzene had no inhibitory effect on phenol, and 2,4-dichlorophenol and *p*NP had stronger antagonistic effects toward the phenol-DmpR complex compared to 3,5-dimethylphenol (Figure 2B).

Analysis of the DmpR structure suggested that the hydroxyl group of phenol plays an important role in binding to DmpR (Park et al., 2020). No inhibitory effects were observed when benzene was added to the phenol and DmpR-GESS reaction system, suggesting that benzene does not bind to the active pocket of DmpR, including its aromatic ring moiety. Phenolic compounds containing functional groups in *meta* or *para* positions, such as 2,4-dichlorophenol or 3,5-dimethylphenol, may bind to the ligand binding site without inducing transcriptional activation, which can suppress the output signal. Among these compounds, *p*NP showed the greatest antagonistic effect and was applied to control the dynamic detection range of the genetic circuit as a resistor for the monitoring of enzyme activity.

### Quantitative Control of the Dynamic Detection Range of a Genetic Circuit Using an Antagonist

**Figure 3:**
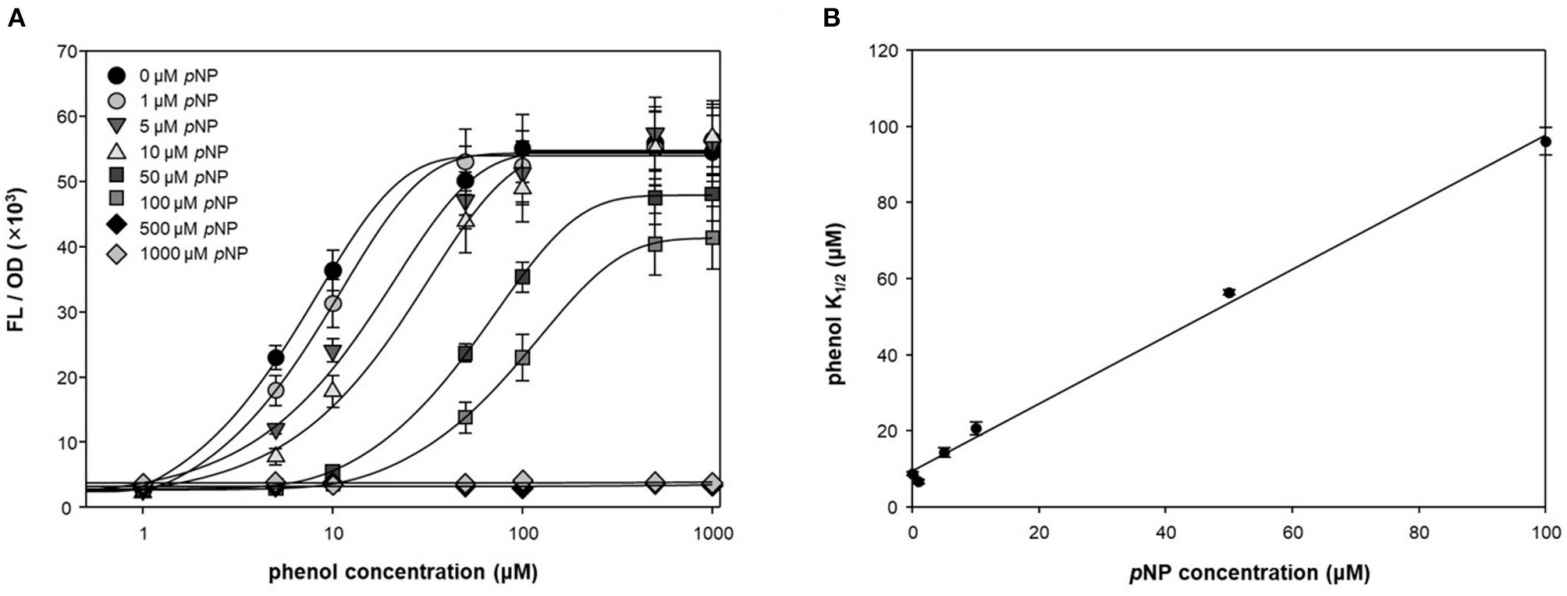
Quantitative suppression of phenol dependent DmpR operator with *p*NP. **(A)** Titration curve of DmpR-GESS was measured at various concentration of phenol and *p*NP. Values represent the means ± SDs of triplicates. **(B)** Phenol concentration of half-maximal fluorescence intensity (phenol K_1/2_) of DmpR-GESS at various concentration of *p*NP. Phenol K_1/2_ is the concentration of phenol at the half-maximal fluorescence signal. Error bars represent the standard deviation of K_1/2_ by four-parameter Hill equation.

To test the antagonistic effect of *p*NP, fluorescence intensities were measured at different concentrations of phenol and *p*NP in a two-step reaction (Figure 3A). *p*NP showed a significant antagonistic effect, even at low concentrations (1 μM). The maximum fluorescence intensity was maintained at concentrations of *p*NP up to 10 μM, but the intensity was lowered at concentrations higher than 10 μM, and the response to phenol was completely lost at 500 μM. The phenol K_1/2_ of DmpR-GESS—the concentration of phenol at the half-maximal fluorescence signal—increased linearly with the *p*NP concentration (Figure 3B). The maximum phenol K_1/2_ of the circuit along with *p*NP increased by ~30-fold compared to in its absence, with a wider dynamic detection range. By adding *p*NP as a GCR, the dynamic detection range of the genetic circuit can be controlled from several μM to hundreds of μM. Therefore, *p*NP is a strong antagonist of DmpR, and can be used to precisely control the genetic circuit.

Phenol and *p*NP are non-metabolites in *E. coli*, and thus they can be maintained at a constant intracellular concentration. Therefore, phenol as a ligand can exhibit high sensitivity, even at low concentrations, and *p*NP as an antagonist can exert a constant inhibitory effect over time. Many aromatic compounds can diffuse freely through the cell membrane via passive transport (Gallert and Winter, 1993; Chen and Fink, 2006). In addition, more than 200 enzymes can generate phenol or *p*NP from phenolic substrates through their catalytic reactions (Kim et al., 2015). Intermolecular release using phenolic substrates and heterologous enzymes in different cells can affect circuit signals in terms of pattern generation or edge detection. By making use of these properties, the circuit can be expanded to function in intercellular quorum sensing.

### Application of Enzyme Inhibitory Effect in the Genetic Circuit

**Figure 4:**
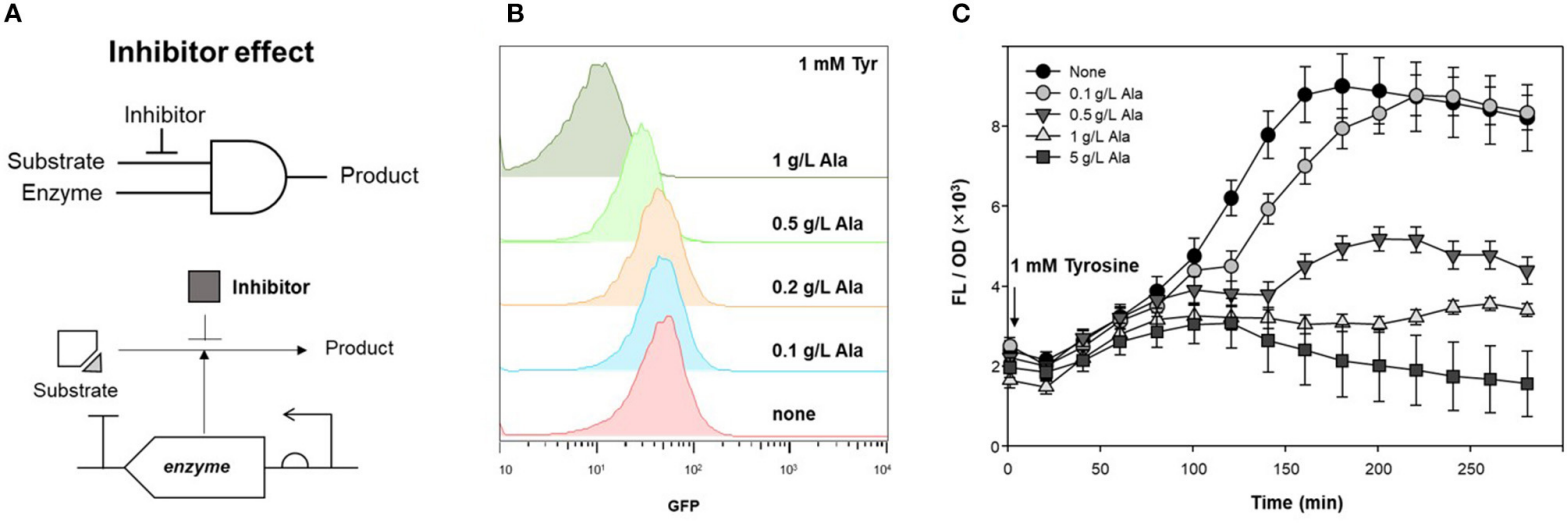
Inhibitory effect of alanine on TPL activity. **(A)** Strategy of competitive inhibitor effect on enzyme in genetic circuit. **(B)** Fluorescence signal control by alanine as an inhibitor for detecting TPL activity using genetic circuit. Flow cytometry profiles of cells harboring pDmpR-GESS and TPL gene at various concentration of alanine. One mM tyrosine was added as a substrate of TPL. **(C)** Time-lapse fluorescence intensity of genetic circuit at various concentration of alanine. Tyrosine (1 mM) was added to detect TPL activity. Values represent the means ± SDs of triplicate measurements.

TPL, which produces phenol from tyrosine as a substrate, was applied to an AND gate of the genetic circuit. To control enzyme expression levels, pAR-TPL was constructed in an L-rhamnose expression system for tight regulation, as TPL can use tyrosine generated by the *E. coli* host's amino acid synthesis pathway (Supplementary Figure 3). Alanine, which is a competitive inhibitor of the TPL beta-elimination reaction, can be used as a GCR for TPL activity (Demidkina et al., 1987). Figure 4A shows the application of the enzyme inhibitor as the resistor in the AND logic gate using an enzyme and its substrate as inputs.

TPL was expressed in LB, after which the cells were transferred to M9 minimal media for two-step induction to maximize fluorescence intensity (Kwon et al., 2020). Figure 4B shows the fluorescence intensity induced by different concentrations of alanine, as measured by flow cytometry. When 1 mM tyrosine was added to M9, the fluorescence intensity, which reflected the activity of TPL, was reduced at concentrations of alanine above 0.5 g/L. For solid phase assays, cells harboring the genetic circuit and TPL gene were incubated in LB agar plate containing 1 mM tyrosine and 1 g/L alanine at 37°C for 20 h. The fluorescence intensity of the colonies was suppressed in LB agar plates when both the substrate and inhibitor were present (Supplementary Figure 4). Thus, alanine can be used as an enzyme inhibitor of TPL and as the resistor in a genetic circuit.

Figure 4C shows the inhibitory effect of alanine in the genetic circuit, measured as the time-lapse fluorescence intensity. The fluorescence intensity was dependent on the inhibitor concentration, and the fluorescence signal was restored at low concentrations of alanine (0.1 g/L). Alanine can be metabolized by the host and, over time, the inhibitory effect may be weakened. In a genetic circuit composed of enzymes, the output can be regulated by reducing the enzyme activity via addition of an inhibitor. If the inhibitor is a metabolite in the host, the intracellular concentration gradually decreases, resulting in a delayed-output signal until enzyme activity is restored.

### Fine Tuning of Genetic Circuit Using Regulator Antagonist and Enzyme Inhibitor

**Figure 5:**
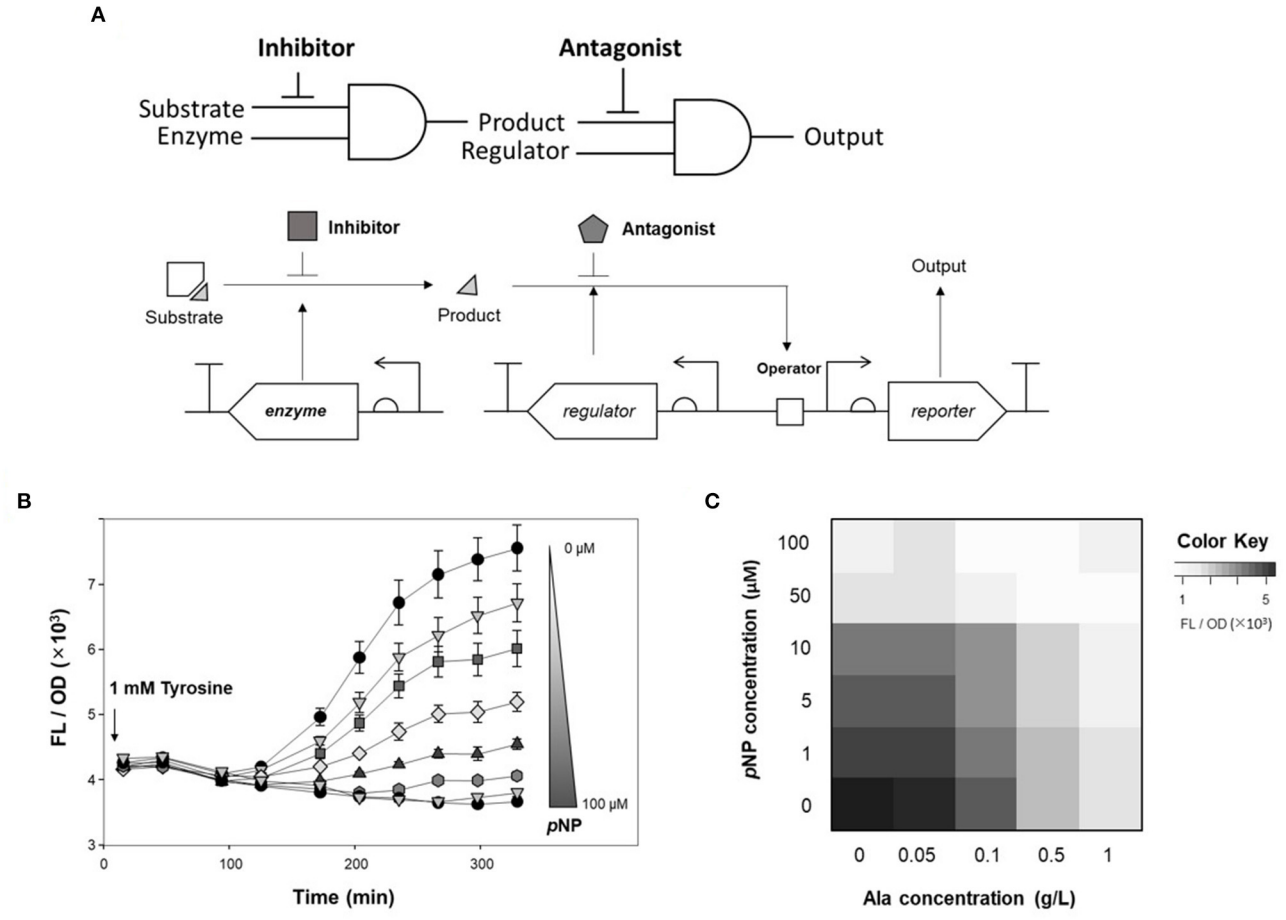
Fine tuning of the genetic circuit using antagonist and inhibitor to detect enzyme activity. **(A)** Schematic diagram of genetic circuit workflow using inhibitors and antagonists. **(B)** Time-lapse fluorescence intensity of GESS harboring TPL gene at various concentration of *p*NP. Values represent the means ± SDs of triplicate measurements. **(C)** Dual inhibitory effect of alanine and *p*NP in fluorescence analysis of TPL activity using the genetic circuit. Values represent the means of triplicate measurements.

To tune the genetic circuit, antagonists and inhibitors were applied to monitor enzyme activity. Of the two AND logic gates of DmpR-GESS (Figure 5A), the first AND gate, consisting of TPL and tyrosine, was able to control the level of product, with alanine used as an inhibitor. As an antagonist, *p*NP can be used to modulate the output signal of the second AND gate using as inputs a regulator and the phenol produced by the first gate.

To quantify the use of *p*NP as a GCR for measuring enzyme activity, time-lapse fluorescence intensity of cells harboring TPL and DmpR-GESS was measured at various concentrations of *p*NP (Figure 5B). The fluorescence intensity decreased as the concentration of *p*NP increased. *p*NP, as a non-metabolite in the host, can maintain its inhibitory effect over time. The antagonist can act as a resistor in the genetic circuit, controlling the output signal through its effects on the regulator.

Finally, alanine and *p*NP as GCRs for each AND gates were applied to control the output of the genetic circuit (Figure 5C). Fine-tuning of the genetic circuit had a greater effect when the two GCRs were applied simultaneously than with a single application. As a result, the genetic circuit can control the output signal when GCRs are added and combined for each logic gates.

One of the great demands of enzyme engineering using genetic circuits is catalytic improvement. Various types of genetic circuits have been developed over the past decade (Eggeling et al., 2015; Mahr and Frunzke, 2016). Many circuit studies investigated the control of the transcriptional signals by replacing genetic elements such as promoters and ribosome binding sites. The dynamic detection range of the genetic circuit is fixed for the original enzyme activity at the beginning of construction. Moreover, the detection range would not be revised during high-throughput screening rounds for better catalysts. Post-translational methods such as the use of protein degradation tags, incorporation of protein-protein interactions, and conformational changes have been used to control the expression of proteins (Pawson and Nash, 2000; Nussinov and Ma, 2012; Cameron and Collins, 2014). However, most of these post-translational methods rely on their specific characteristics for each new property of a genetic circuit.

To apply GCR to the design of genetic circuits, small molecules should be transported into the cell by passive or active transport systems. Given this basic condition, the output signal can be controlled by adjusting the activity of the enzyme or sensitivity of the regulator. The use of a GCR enables screening for higher enzyme activity, or to control a dynamic sensing and regulation system which can generate multiple outputs using a single module.

## Conclusions

This study demonstrates an approach for controlling genetic circuit properties using small molecules which interact with the enzyme and regulator which are parts of the genetic circuit. The enzyme and regulator generally work with specific small molecules, and these biochemical reactions are inhibited by other substances with similar molecular properties and act as inhibitors and antagonists. In this study, alanine was used to inhibit TPL and *p*NP was used as an antagonist of DmpR to produce a genetic circuit for monitoring enzyme activity. These inhibitory small molecules, by interacting with the enzyme or regulator, can be used as logical elements for circuit design. Our approach may be applied to a range of genetic circuit-mediated enzyme applications, such as the directed evolution of enzymes, without the need for reconstructing each different genetic circuit.

## Data Availability Statement

The raw data supporting the conclusions of this article will be made available by the authors, without undue reservation.

## Author Contributions

S-GL conceived the study. ER performed the plasmid construction. KK performed the fluorescence analysis. JL performed the fluorescence image. S-GL, KK, HK, S-JY, HL, and D-HL wrote the manuscript. All authors have given approval to the final version of the manuscript.

## Conflict of Interest

The authors declare that the research was conducted in the absence of any commercial or financial relationships that could be construed as a potential conflict of interest.
