# Supplementary material for: Antagonistic Control of Genetic Circuit Performance for Rapid Analysis of Targeted Enzyme Activity in Living Cells
Source: Front Mol Biosci. 2021 Jan 12;7:599878. doi: 10.3389/fmolb.2020.599878 (PMC7835892; doi:10.3389/fmolb.2020.599878)

Supplementary Material

Antagonistic control of genetic circuit performance for rapid analysis of targeted enzyme activity in living cells

Kil Koang Kwon^1^, Haseong Kim^1,2^, Soo-Jin Yeom^3^, Eugene Rha^1^, Jinju Lee^1,2^, Hyewon Lee^1^, Dae-Hee Lee^1,2^ and Seung-Goo Lee^1,2,*^

# Supplementary figures

## Fig. S1.

Fluorescence intensity of genetic circuits using mutant and wild-type DmpR against phenol and *para*-nitrophenol. Values represent the means ± SDs of triplicates.


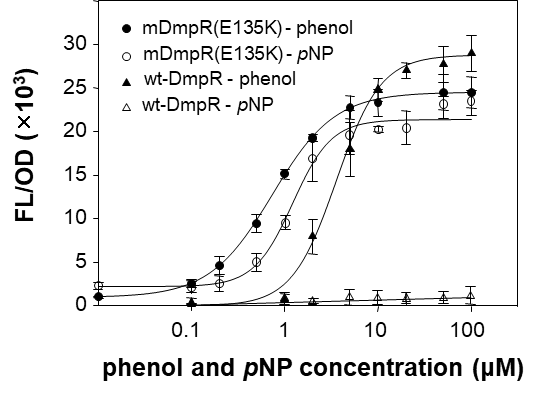


## Fig. S2.

DmpR sensory domain dimer (PDB: 6IY8). The a/b dimer was superimpose on the b/a dimer. The a/b (holo form) and b/a (apo form) dimer cartoons were indicated by ribbon and wire, respectively. In a/b and b/a, Glu135 and Arg36 were marked with green and orange ball and sticks. Phenol was represented as CPK.


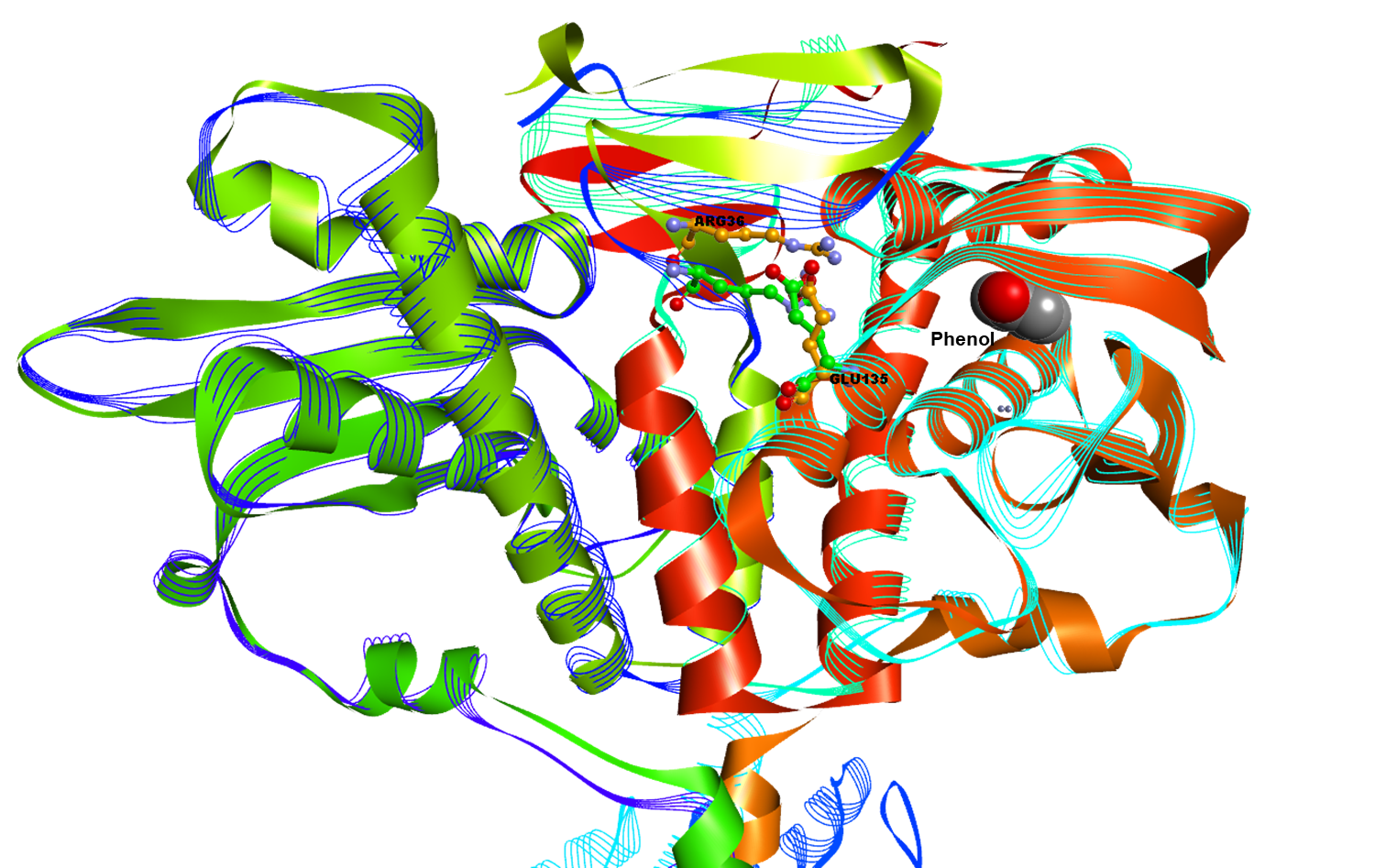


## Fig. S3.

Fluorescence intensity of TPL-GESS at various concentration of rhamnose for control of enzyme expression.


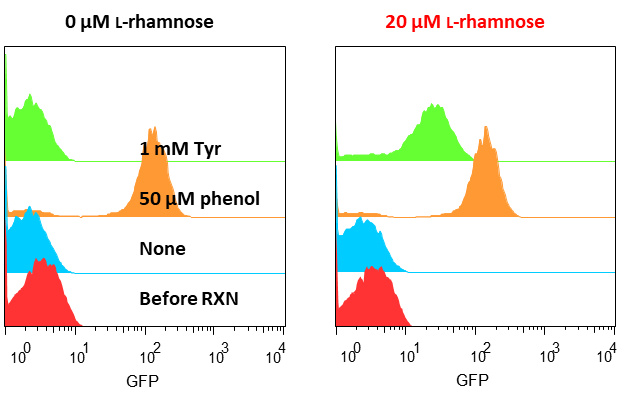


## Fig. S4.

Fluorescence image of DmpR-GESS cell harboring TPL gene on LB agar plate containing tyrosine and alanine.


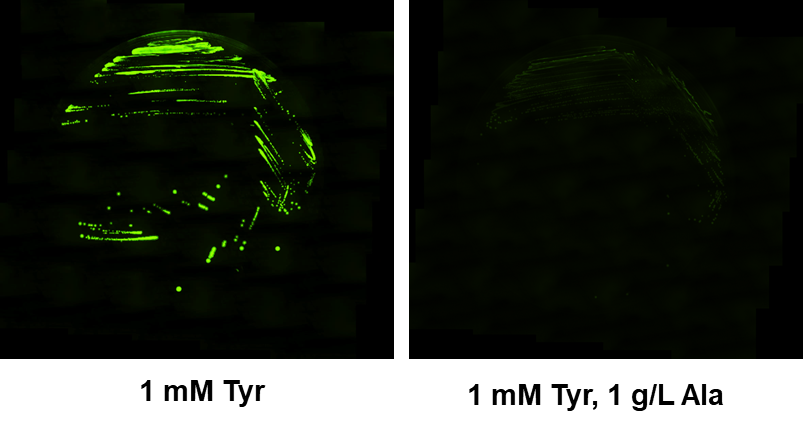

Supplement: Supplementary file 1 [file Data_Sheet_1.docx]
